# Designing Cases for Case-Based Immunology Teaching in Large Medical School Classes

**DOI:** 10.3389/fimmu.2020.00995

**Published:** 2020-05-27

**Authors:** Jeffrey P. Novack

**Affiliations:** College of Medicine, Pacific Northwest University, Yakima, WA, United States

**Keywords:** immunology education, case-based learning, medical school education, team-based learning, key concepts, active learning, integrated curriculum

## Abstract

Case-based, interactive sessions for small groups (in a large medical school class of 150 students) reinforces basic immunology concepts by including clinical scenarios that stimulate student learning and consolidate critical concepts. Careful design of cases (designing backwards from the key concepts) leads students through successively more complicated and linked group-work questions. This paper details why cases are effective learning tools, how to design an effective case, how to ask appropriate questions and how to help students apply basic immunology concepts to a case. Each group work session is facilitated and followed by a question and answer presentation by faculty, where student groups are directly asked to answer the questions and also challenged with “bonus questions” not presented with the original case. This allows students to “put together” immunology information into a “story” that they can tell and prevents student frustration by summarizing the results at the end of each case. Case design is carefully discussed including clinical relevancy and accuracy, how to write questions that do not give away the answers, how to emphasize mechanistic questions that allow students to “clinically explain as a physician” the immunological basis for the answers. Additionally, students better understand the role of immunity in both normal and disease states. A case-based approach promotes student learning by re-emphasizing basic concepts in the context of the case and promotes better students understanding of critical immunological concepts.

## Introduction

Previous studies have shown that problem-based learning can be effective in large medical school classrooms (1). A meta-analysis of active learning studies (2) in undergraduates showed an advantage for student learning and satisfaction for specific active learning activities over traditional lectures in the sciences. However, the literature on active learning may also have some publication bias (3), but the evidence in the literature still strongly indicates the value of interactive learning activities and the overall effectiveness of problem-, team- and case-based learning.

Medical schools are tending to increase active learning in the curriculum and also to integrate basic science information with clinical cases across disciplines (4). In addition, medical educators have proposed using Entrustable Professional Activities (EPAs) for undergraduate medical education, similar to how these are used in Graduate medical education (5). Case-based learning is especially appropriate for pre-clinical training in undergraduate medical education. A randomized study looked at case-based learning and found it comparable to problem-based learning with better student satisfaction (6). Burgess et al. (7) examined team-based learning (TBL), that uses fewer faculty than the problem-based learning (and therefore may be easier to implement for many medical schools) and found that TBL fostered more competitiveness and desire to learn, but that PBL yielded increased clinical reasoning. The authors concluded that some hybrid of these two approaches might yield the best results. A subsequent study showed that students preferred the team based approach to problem based learning, but student preference is not always indicative of actual student learning (7).

Medical cases stimulate students to be more active in their learning and to not just memorize facts, but to attempt to learn critical concepts. Chonkar et al. (8) have hypothesized that students who participate in case-based learning gain deeper and more long lasting knowledge than students who seek to mostly memorize (but not apply) critical scientific facts. Case based learning has been shown to motivate students to learn more deeply in a number of studies (9). Turk et al. (10) showed a significant improvement in practical knowledge application (OSCE scores) for case based learning over the traditional approach. Given the widespread use of case studies, the evidence for increases in student motivation, the opportunities for deeper and more long-lasting learning and the increased ability to apply case based concepts to practical applications, this paper attempts to describe a method for designing better cases that are more applicable to medical student needs.

In order to better incorporate active, case-based learning into the curriculum, medical school educators need cases that ask carefully designed questions that challenge but do not frustrate students. Problem based learning (on which case based learning is based) helps stimulate student inquisitiveness, but one of the possible drawbacks is student frustration. Cases need be designed to emphasize critical learning objectives, to be medically relevant and to not easily give away the answer to promote differential diagnosis skills. Also, using the “backward” design approach for medical school cases (11) helps to insure that the learning objectives are covered in the case. The steps for designing a medical school case are outlined below, using an actual case and the questions for the case as examples to help illustrate medical school case-based design and implementation.

Specifically, for immunology education in medical school, using team-based learning has been shown to be an effective method (12). While our approach for case based learning uses some of the team based approaches (such as pre-class learning modules (combined lecture and self-guided learning, with learning objectives embedded in the learning materials), there was no readiness assurance testing. Instead, students were motivated to learn because they knew their group could be called on to explain their answers.

## Medical School Classroom Set-Up for Case-Based Learning

These cases are designed for students working in groups of 4–6 students in a large classroom (one class of 150 students, or two sessions of 75 students each). Groups size was 4–5 students from the literature for the effectiveness of small groups and because our medical school had preassigned groups of four to five students (13). About 3–5 faculty “advisors,” who have been prepped on the case and have an answer key, circulate in the room (it can be a large lecture hall, but a smaller room with moveable desks works better) to ask questions and guide, but they do not directly answer question or explain (Student: What is the answer to this question? Faculty: What do you think the lab result from the case shows and why is it important?). The students have access to the case (with the questions) at least 3–5 days in advance of class and are required to answer a few very basic questions individually on the case before the class session (What is the case about? What is your differential diagnosis?). Students can use any available resources to answer the basic questions or the more detailed questions in class and are encouraged to use material from lecture, from pre-recorded lectures of from faculty directed studies and readings. Lecture material and reading material all have learning objectives to help guide students in their preparation for the in-person classroom activity. Student groups get an allotted time in class to answer the questions, and then the class reconvenes and faculty ask questions of the groups. The groups are called on randomly to answer the questions in the case and faculty can ask additional “bonus” questions, designed to test knowledge of concepts that are NOT on the case the students are given. One advantage of allowing students to use any resources, including the internet, is that students will quickly gravitate to more reliable resources, if they know they have to defend their answers. For an incorrect answer, a faculty facilitator can ask where the student obtained that information, in order to understand whether the misinformation came from a source or from the student not understanding the question or the material. If a group has an incorrect answer or cannot come up with an answer, the faculty facilitator can ask some leading questions or ask if another group can offer assistance or call on another group to help out. If students are still struggling with a particular question, further leading questions can be asked of the entire class. Usually, the simpler questions are asked first and the more difficult questions come at the end of a case. About three to five cases with questions can be covered in a 2 h block with a quiz at the end. The quiz is often based on some of the key learning objectives covered in the case-based session.

## Backwards Design of a Medical School Case

For backwards design of a case, it is important to start with the learning objectives and core competencies that we want the students to have (14). This helps insure that the case covers the critical learning concepts we want the students to encounter. We also deliberately integrate a number of topics (for this case, immunology, microbiology, pathology, pharmacology, physical exam results, and lab medicine are integrated) in order to have students apply prior knowledge and make the case more realistic.

For this pre-clinical medical school case, the learning objectives were the following:

1. Describe the local and systemic effects of gram-negative bacterial sepsis including expected physical exam and lab results.

2. Explain the molecular mechanisms of how gram negative bacterial infection and septic shock causes elevated body temperature, chills, elevated CRP (C-reactive protein), elevated white cell count, neutrophilia, increased band cells, and hypotension.

3. Explain why high doses of the appropriate anti-bacterial agents can cause increased shock symptoms and possible death in septic shock.

In this example, the learning objectives help set up the case. The case has to be a gram negative septic shock case. The physical exam, lab results, CBC and other tests should reflect a patient in gram negative sepsis. Before starting the case, however, it is helpful to design some of the open-ended questions to ask. The questions start off simply from basic concepts and help to lead the students through the basic concepts without giving away too much and then get to the more difficult applications of these concepts (see Table 1).

**Table 1:** Immunology Concept Mapping Chart.

**Core immunology concept**	**Learning objective**	**Question on case:**
PAMP and DAMP activation of innate immunity	Describe the TLRs that recognize specific PAMPs and DAMPs.	What Toll-like receptors (TLRs) are likely to be activated by this infection?
TLR activation and signaling	Explain how TLR activation/signaling primes the innate immune response	What cytokines would you expect to be elevated in the blood in this patient after this infection?
Actions of mediators of acute inflammation	Describe the actions of mediators acute inflammation (IL-1/TNF-alpha	Why is the white count elevated? What cell type is most likely to be elevated? Why are the band cells increased? What is the molecular mechanism for the increase on neutrophils and band cells
Physiological and effects of acute inflammation.	Describe the physiological effects of elevated IL-1, TNF-alpha and IL-6.	What is the significance of the increasing fever and the presence of bacteria in the blood? What is the molecular mechanism for the fever increase and why does this concern you?
Pathological effects of acute inflammation.	Describe the pathological effects of elevated IL-1, TNF-alpha and IL-6.	Why is the blood pressure dropping in this patient? Why is does he have systemic edema? What are the molecular mechanisms for the drop in blood pressure?

Other helpful ways to design and write a case include modeling the case after a real case, following the SOAP note format (Subjective, Objective, Assessment, Plan (and leaving OUT the Plan from the case), and setting up the case in a specified manner:

1. Age, gender how, and where the patient presents.

2. Subjective: Chief complaint, relevant history (how long have the symptoms been present, any other relevant history, travel, operations, medical history).

3. Objective (where relevant): Vital signs, physical exam results, lab tests results, relevant drugs the patient is taking.

4. Assessment: Do NOT give a diagnosis, let the students do this, but ask the questions.

5. Plan: leave the plan out of the case, but ask the students questions about the plan or treatments.

6. Check the case for accuracy when done, consult with a clinical colleague with expertise. Are there any other explanations for the case that could lead the students to alternative explanations? Sometimes the case can be intentionally vague, and we can ask for further tests to help distinguish between possible diagnoses.

7. Avoid Zebra cases: Zebra cases are cases with rare genetic defects (many of the currently available immunology cases are zebra cases) and these cases often will not be seen by most students and represent a small specific area of immunology. Feedback from clinical site preceptors has indicated that students need additional basic science applications in more common cases. Medical boards also ask questions on these rare cases. Designing more common cases makes it more clinically relevant for the students and we make charts for the more rare genetic defects in immunology for use in board studying.

8. Keep the vignette (case) short and to the point.

9. Instead of creating an entirely new case, it sometimes is effective to change one or more test results to point the case toward a different diagnosis. The question can then be asked: If the test results are now Y instead of X, what is the explanation?

Sample case (student version):

A 62-years old male initially presents with fever (38.5°C), elevated C-reactive protein (CRP) levels and ESR (erythrocyte sedimentation rate), hypotension (BP 100/70), white count (12,500 and 15% bands), hyperglycemia (blood glucose, 140), and lower right flank pain. He has had a previous history of benign prostatic hyperplasia and a urinary bladder catheterization (but no history of diabetes). Two days after the last catheter insertion, he developed a fever and now on the third day he is mildly disoriented, and on examination has tenderness in the lower right quadrant. His urine culture yields over 102 gram negative rods. Twenty-four hours later, the patient's condition has deteriorated. His blood pressure has dropped to 85/65 (is requiring a crystalline blood infusion), his blood glucose is 150, he has systemic tissue swelling, his CRP has increased, and his temperature is now increased to 39°C. All three blood samples currently yield multiple colonies of gram negative bacteria on culture.

## Designing the Questions for Case

When designing the questions for a medical school case, it is important to let the students do the differential diagnosis. Another key to designing the questions is to start with the simpler questions, to help lead the group toward the more complex explanations. Also, asking questions about the molecular mechanisms is particularly important for understanding the immunological basis for the response. For the case above, here are some sample questions and the rationale for the questions. Students get the questions in italics along with the case. Bonus questions are not included in the preview that the students get, but are asked of the group during the class by faculty facilitators:

1. What Toll-like receptors (TLRs) are likely to be activated by this infection? What cytokines would you expect to be elevated in the blood in this patient after this infection?

These questions start with the initial mechanism of gram negative bacterial infections, how they trigger TLR2 and TLR4 activation. The student should recognize that TLR4 is specific for LPS and gram negative bacteria. Also, the goal is to have students groups start with the bacterial infection, go to the TLRs, then the cytokines and then the local and systemic effects. So the first question is simple and leading question designed to set up the subsequent questions and help the students groups think in a linear progression.

*2. Initially, this patient has local tenderness and flank pain. Explain what cells you expect to be increased locally (in the bladder and kidney) in the first 24 h? What is the molecular mechanism for these cells migrating to the initial site of infection?* This question focuses the student groups on the molecular mechanisms for the induction of neutrophil and leukocyte rolling (Selectins) and tight binding (chemokine inside-out signaling and high affinity integrin binding) and migration. The student should recognize that neutrophils will be the first immune cells to migrate to an area of inflammation, followed by macrophages and then lymphocytes and should be able to elucidate the stepwise mechanism of leukocyte migration described above.

Bonus questions (Not on the student copy): If a patient has a defect in neutrophil migration, would that increase susceptibility to infectious agents and what specific agents?

This bonus question addresses the role of neutrophils in protecting from bacterial infections. Lack of neutrophils at the site of an infections would decrease the response to bacterial infections and particularly to infections with Staphylococcus or Streptococcus on the skin.

3. What is the significance of the increasing fever and the presence of bacteria in the blood? What is the molecular mechanism for the fever increase and why does this concern you?

This question focuses the students groups on explaining the physical exam results and the role of inflammatory cytokines (pyrogens) in fever. Also, it helps students trace the course of sepsis and to understand the clinical effects and danger to the patient. The student should be able to start with TLRs, go to increased cytokines (IL-1 and TNF-alpha, increased prostaglandins in the hypothalamus and then to increased temperature set point. An increasing fever indicates the infection is getting worse, not better (point out that the temperature should be taken at a similar time since body temperature can vary with circadian rhythms).

4. Why is the white count elevated? What cell type is most likely to be elevated? Why are the band cells increased? What is the molecular mechanism for the increase on neutrophils and band cells? What do increases C-reactive protein and ESR (Erythrocyte Sedimentation Rate) mean and why are they increased?

These questions address the specific laboratory and physical exam results that indicate sepsis. Student groups should be focused on the molecular mechanisms for the CBC, leukocytosis, neutrophilia and the increase in band cells results in the case. Students should be able to describe the increase in inflammatory cytokines (IL-1, TNF-alpha, IL-6) the increase is CRP (from the liver), the increase in G-CSF and GM-CSF that will increase band cells (immature neutrophil) production and release from the bone marrow.

*Bonus question: In a patient undergoing chemotherapy for cancer who has neutropenia, what drug could be given to increase the absolute neutrophil count*?

This bonus question is designed to help the student groups think about the role of G-CSF and GM-CSF in responding to infections. Usually G-CSF (peg-filgrastim, Neupogen, or a similar drug) is given to increase neutrophils. It enhances both neutrophil production and release. GM-CSF is also effective but is used less often.

5. *Why is the blood pressure dropping in this patient? Why is does he have systemic edema? What are the molecular mechanisms for the drop in blood pressure?* These questions help to focus the student on the clinical situation of septic shock and to understand the underlying immunological mechanisms for the clinical effects. Blood pressure drops because fluid (exudate) leaks out permeable blood vessels in shock (high levels of IL-1 and TNF-alpha). Additionally, cardiac output is diminished. Systemic edema is due to the systemic IL-1 and TNF-alpha, the increased permeability throughout the circulatory system and the formation of exudate in the extracellular space.

*Bonus question: What is a possible danger of giving a high dose of antimicrobial therapy in this case*?

Why do a bonus question? The bonus question is designed to keep the after group-work questioning “fresh,” since the students know that may get MORE questions than just what are specified on the question sheet and to help students think “on the spot” more.

This bonus question is designed to help the student groups understand the molecular mechanism of LPS-induced septic shock and to understand the possible role of antimicrobials in releasing more LPS (due to bacterial killing) and making shock worse. It aligns with the learning objectives from the pre-class material.

Sometimes, these questions may call for a differential diagnosis and while these are first or second year medical students, it is meant to help them practice this skill in a low stakes environment. Also, the review helps to model the thinking used for a differential diagnosis.

## Summary

Using cases in a group, active learning context to highlight the basic science concepts in immunology is a useful tool for engaging medical students and for consolidating their knowledge in immunology. Careful design of cases helps prepare students for clinical preceptorships in their third and fourth years. Additionally, these cases can help integrate a number of subject areas including immunology, microbiology, pathology, lab medicine and internal medicine. The sample case presented above is an example of a possible way to design cases. The “backwards” engineering of the case from learning objectives and the clinical relevance of the case helps to make this case-based learning more effective for the students.

## Author Contributions

The author confirms being the sole contributor of this work and has approved it for publication.

## Conflict of Interest

The author declares that the research was conducted in the absence of any commercial or financial relationships that could be construed as a potential conflict of interest.
